# The Reduction of PSMB4 in T24 and J82 Bladder Cancer Cells Inhibits the Angiogenesis and Migration of Endothelial Cells

**DOI:** 10.3390/ijms25105559

**Published:** 2024-05-20

**Authors:** Yi-Hsuan Lin, Tzu-Min Chen, Yu-Ling Tsai, Wen-Chiuan Tsai, Hisao-Hsien Wang, Ying Chen, Sheng-Tang Wu

**Affiliations:** 1 Department of Biology and Anatomy, National Defense Medical Center, Taipei 11490, Taiwan; lyhbs14@gmail.com (Y.-H.L.); ctm999322@gmail.com (T.-M.C.); 2 Department of Pathology, Tri-Service General Hospital, National Defense Medical Center, Taipei 11490, Taiwan; c909228@gmail.com (Y.-L.T.); ab95057@hotmail.com (W.-C.T.); 3 Department of Urology, Cheng Hsin General Hospital, Taipei 11490, Taiwan; hsiao386@ms13.hinet.net; 4 Division of Urology, Department of Surgery, Tri-Service General Hospital, National Defense Medical Center, Taipei 11490, Taiwan

**Keywords:** bladder cancer, PSMB4, migration, angiogenesis, tube formation

## Abstract

Bladder cancer (BC) is a malignant tumor of the urinary system with high mortality and recurrence rates. Proteasome subunit type 4 (PSMB4) is highly expressed and has been identified as having oncogenic properties in a variety of cancer types. This study aimed to explore the effect of PSMB4 knockdown on the survival, migration, and angiogenesis of human bladder cancer cells with different degrees of malignancy. We analyzed the effects of PSMB4 knockdown in bladder cancer cells and endothelial cells in the tumor microenvironment. PSMB4 was highly expressed in patients with low- and high-grade urothelial carcinoma. Inhibition of PSMB4 reduced protein expression of focal adhesion kinase (FAK) and myosin light chain (MLC), leading to reduced migration. Furthermore, the suppression of PSMB4 decreased the levels of vascular endothelial factor B (VEGF-B), resulting in lower angiogenic abilities in human bladder cancer cells. PSMB4 inhibition affected the migratory ability of HUVECs and reduced VEGFR2 expression, consequently downregulating angiogenesis. In the metastatic animal model, PSMB4 knockdown reduced the relative volumes of lung tumors. Our findings suggest the role of PSMB4 as a potential target for therapeutic strategies against human bladder cancer.

## 1. Introduction

Bladder cancer (BC) is a common malignant tumor of the urinary system, one of the ten most common malignant tumors in the world, and the fourth most common malignant tumor in men, with high morbidity and mortality [1,2]. Although the five-year survival rate is 77 percent for patients with bladder cancer, 5 percent of patients exhibit metastatic disease [3]. Bladder cancer is divided into two types—non-muscle-invasive bladder cancer (NMIBC) and muscle-invasive bladder cancer (MIBC)—based on tumor invasion into the muscular layer of the bladder. NMIBC accounts for approximately 75% of newly diagnosed bladder cancers. In addition, the postoperative recurrence rate of patients with NMIBC is 50% to 70% [4]. However, nearly 15% of NMIBC tumors eventually penetrate the basement membrane barrier and develop into MIBC [5].

To support tumor cell growth and metastasis, the vascular network provides oxygen and nutrients in the hypoxic tumor environment [6]. In the hypoxic environment, hypoxia-inducible factor 1α (HIF-1α) stimulates the expression of downstream vascular endothelial factor (VEGF) and nitric oxide synthase (NOS) [7]. The interaction of VEGF and VEGFR2 in vascular endothelial cells increases angiogenesis [8,9]. The PI3K/AKT/mTOR signaling pathway also regulates the nitric oxide (NO) content in endothelial cells to facilitate angiogenesis [10]. VEGF and its regulation of the expression of angiogenesis-related factors play an important role in the occurrence and development of bladder cancer.

The proteasome is a complex composed of multiple subunits [11]; the ubiquitin–proteasome system (UPS), together with ubiquitination signaling, is responsible for degrading misfolded or damaged intracellular proteins [12]. Proteasome subunit type 4 (PSMB4), also known as 20S proteasome subunit β-7, is one of the important subunits that contributes to the complete assembly of the 20S proteasome complex [13]. Studies have shown that PSMB4 is highly expressed in a variety of cancer types, such as breast cancer [14], brain glioma [13], ovarian cancer [15], and myeloma [16]. However, the role of PSMB4 in bladder cancer remains unclear. This study aimed to explore the effect of PSMB4 knockdown on the survival, migration, and angiogenesis of human bladder cancer cells with different degrees of malignancy.

## 2. Results

### 2.1. Analysis of PSMB4 Expression in Patients with Bladder Cancer and Establishment of A PSMB4 Silencing System in Bladder Cancer Cells

**Figure 1:**
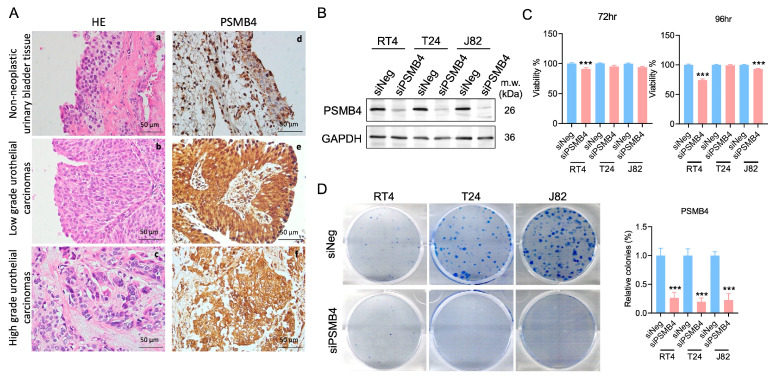
PSMB4 expression in patients with bladder cancer and bladder cancer cell lines. (**A**) a–c: Nonneoplastic urinary bladder tissue; low-grade and high-grade urothelial carcinoma tissues (H&E, 400×). d–f: Immunohistochemical staining of PSMB4 in nonneoplastic urinary bladder tissue and in low-grade and high-grade urothelial carcinoma tissues (PSMB4, 400×). Scale bar = 50 μm. (**B**) PSMB4 protein expression after siPSMB4 transfection for 72 h in RT4, T24, and J82 cells (*n* = 10). (**C**) The viability of RT4, T24, and J82 human bladder cancer cells was evaluated using an MTT assay after PSMB4 silencing for 72 and 96 h (*n* = 6). (**D**) Colony formation assay of bladder cancer cells after PSMB4 downregulation (*n* = 5). Data are presented as the mean ± SEM. *** *p* < 0.001 compared to the siRNA negative control group, determined by unpaired *t*-test with the Mann–Whitney test.

Analysis of a bladder carcinoma tissue array showed that the expression of PSMB4 was higher in tissues from patients with low- and high-grade urothelial carcinoma than in nonneoplastic bladder tissues (Figure 1A). To investigate the role of PSMB4 in bladder cancer, the NMIBC cell line RT4 and the MIBC cell lines T24 and J82 were used for siPSMB4 transfection to inhibit PSMB4 expression (Figure 1B). PSMB4 inhibition decreased the viability of RT4 cells after siPSMB4 transfection for 72 and 96 h as well as that of J82 cells after 96 h of transfection. (Figure 1C). We assessed the long-term clonogenic survival of RT4, T24, and J82 cells after the silencing of PSMB4 expression. The results showed that the number of colonies was decreased after PSMB4 knockdown (Figure 1D).

### 2.2. Silencing PSMB4 Reduced the Migration Ability of Bladder Cancer Cells

**Figure 2:**
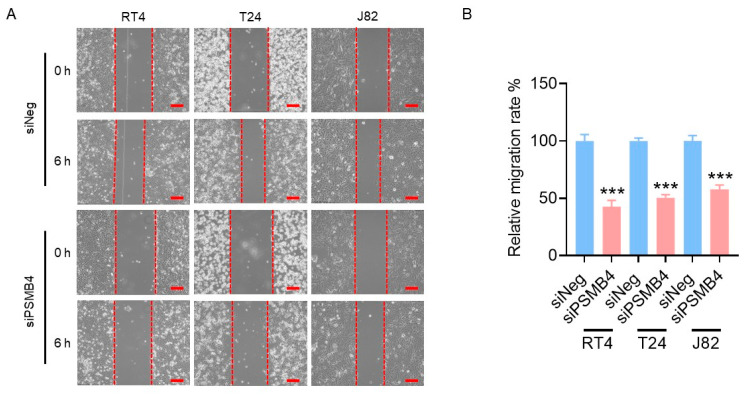
The migration ability of bladder cancer cells after PSMB4 silencing. (**A**) The migration ability of RT4, T24, and J82 human bladder cancer cells was measured via a wound healing assay. Cells were incubated with siPSMB4 for 72 h; then, wounds were created by scratching the cell layer with a P200 pipette tip before observing them for 6 h. Scale bar = 200 μm. (**B**) Quantification of the relative migration rate. Data are presented as the mean ± SEM (*n* = 5). *** *p* < 0.001 compared to the siRNA negative control group, determined by unpaired t-test with the Mann–Whitney test.

**Figure 3:**
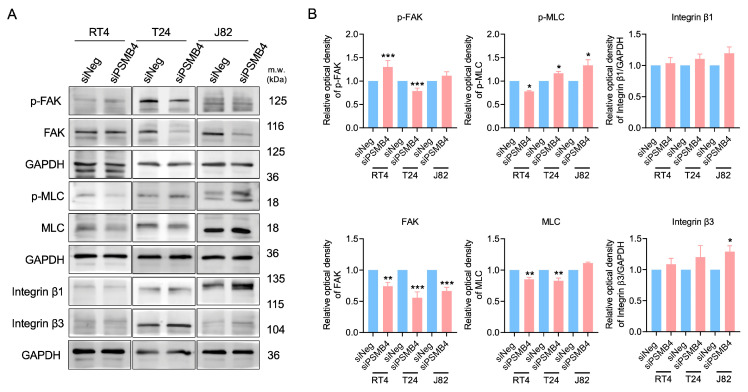
The levels of migration-related proteins in bladder cancer cells after PSMB4 knockdown. (**A**) The protein levels of FAK, p-FAK, integrin β1, integrin β3, MLC, and p-MLC in RT4, T24, and J82 cells after treatment with siPSMB4 for 72 h were measured by Western blotting. We used GAPDH as the loading control. (**B**) The relative quantification of the aforementioned proteins. Data are presented as the mean ± SEM (*n* = 6). * *p* < 0.05, ** *p* < 0.01, and *** *p* < 0.001 compared to the siRNA negative control group, determined by unpaired t-test with the Mann–Whitney test.

Compared with the siNegative (control) groups, cell migration was significantly decreased (by 50%) in the siPSMB4 groups of RT4, T24, and J82 cells (Figure 2). Next, adhesion-related proteins were analyzed. The level of phosphorylated FAK was increased but FAK, p-MLC, and MLC levels were decreased after PSMB4 knockdown in RT4 cells. In T24 cells, after silencing PSMB4, the p-MLC level was increased but the p-FAK, FAK, and MLC levels were reduced. In J82 cells, after silencing PSMB4, the p-MLC and integrin β3 levels were increased, although the FAK level was decreased (Figure 3). These results suggested that PSMB4 inhibition reduced the migration ability of human bladder cancer cells, which may be mediated by adhesion-related proteins.

### 2.3. Knockdown of PSMB4 Inhibited HUVEC Angiogenesis

**Figure 4:**
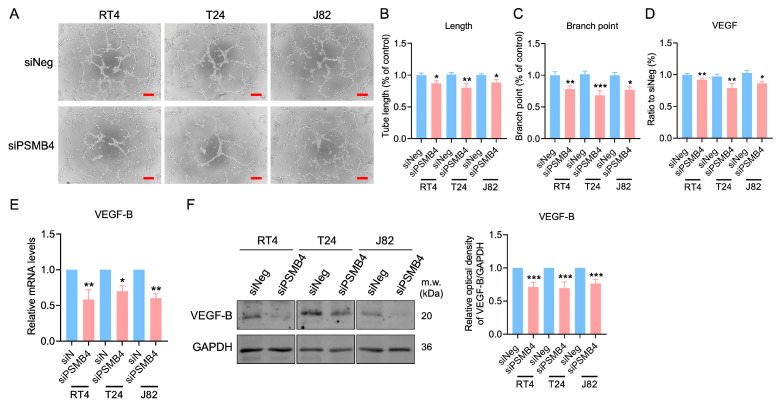
The effect of PSMB4 knockdown on HUVEC angiogenesis. (**A**) The conditioned medium of RT4, T24, and J82 cells transfected with siPSMB4 was collected. HUVECs were cultured with these conditioned media for 6 h. The tube formation ability was evaluated. Scale bar = 200 μm. (**B**,**C**) The length and branching of formed tubes were analyzed (*n* = 5). (**D**) The VEGF protein content in the conditioned medium of RT4, T24, and J82 cells after treatment with siPSMB4 was measured with an ELISA kit (*n* = 4). (**E**) The mRNA levels of VEGF-B in RT4, T24, and J82 cells after treatment with siPSMB4 for 72 h were measured via real-time PCR (*n* = 4). (**F**) VEGF-B protein expression after siPSMB4 transfection for 72 h in RT4, T24, and J82 cells (*n* = 7). Data are presented as the mean ± SEM. * *p* < 0.05, ** *p* < 0.01, and *** *p* < 0.001 compared to the siRNA negative control group, determined by unpaired t-test with the Mann–Whitney test.

**Figure 5:**
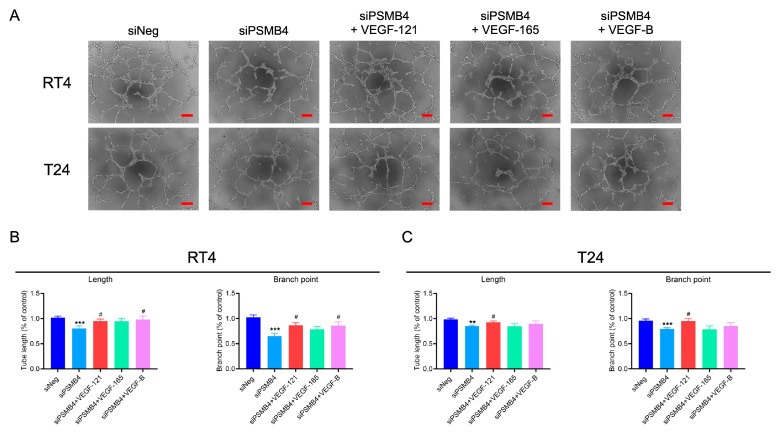
The role of VEGF factors in PSMB4 knockdown in HUVEC angiogenesis. (**A**) The conditioned media from RT4 (*n* = 7) and T24 (*n* =11) cells transfected with siPSMB4 were collected. The angiogenesis factors of VEFG-121, VEGF-165, and VEGF-B (2 μg/mL) were added into the condition media. Tube formation was observed after 6 h. (**B**,**C**) The length and branching of formed tubes in RT4 and T24 cells were analyzed. Scale bar = 200 μm. Data are presented as the mean ± SEM. ** *p* < 0.01, and *** *p* < 0.001 compared to the siRNA negative control group. # *p* < 0.05 compared to the siPSMB4 control group, determined by unpaired t-test with the Mann–Whitney test.

HUVECs were cultured with the siPSMB4 conditioned media, and the lengths and branch numbers of the formed tubes were analyzed. Compared with the control group, the tube length and branch numbers were decreased in the siPSMB4 group (Figure 4A–C). The VEGF content was decreased in conditioned medium from siPSMB4-transfected cells (Figure 4D). The mRNA expression of VEGF-B was downregulated in RT4, T24, and J82 bladder cancer cells (Figure 4E). Moreover, the VEGF-B protein expression level was also decreased after siPSMB4 transfection (Figure 4F). Although VEGF-A mRNA expression was reduced in T24 and J82 cells, there was no significant difference in the VEGF-A protein expression level. While the expression level of the VEGF-C protein was decreased in RT4 cells, it was not significantly different in T24 and J82 cells (Supplementary Figure S1). To elucidate the role of VEGF factors in PSMB4 knockdown on HUVEC angiogenesis, we added VEGF factors includingVEGF-121, VEGF-165, and VEGF-B into the siPSMB4 conditioned media (Figure 5A). The results indicated that VEGF-121 could revert the tube length and branch numbers back to basal levels in both RT4 and T24 cells (Figure 5B,C). VEGF-B could reverse the tube length and branch numbers in the siPSMB4 group of RT4 cells (Figure 5B). These results show that the amount of VEGF-B secreted from silenced bladder cancer cells was lower than that secreted from control cells, which may contribute to the inhibition of angiogenesis.

### 2.4. Knockdown of PSMB4 Inhibited the Migration of HUVECs

**Figure 6:**
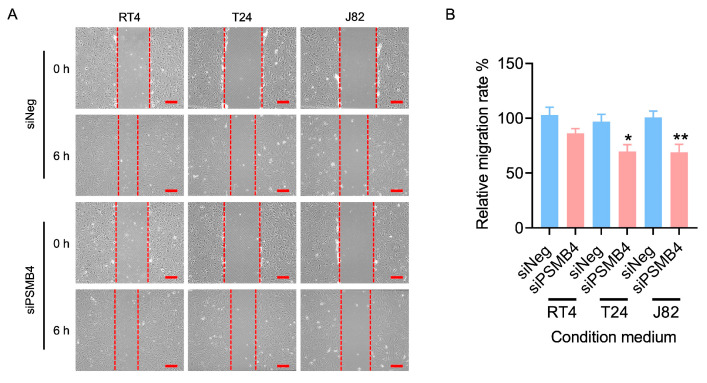
The effect of PSMB4 knockdown on HUVEC migration. (**A**) The conditioned media of RT4, T24, and J82 cells transfected with siPSMB4 were collected. HUVECs were incubated with conditioned medium; then, wounds were created in the cell layer by scraping with a P200 pipette tip before observing them for 6 h. The migration ability of HUVECs was measured using a wound healing assay. Scale bar = 200 μm. (**B**) The quantitative relative migration rate is shown. Data are presented as the mean ± SEM (*n* = 11). * *p* < 0.05 and ** *p* < 0.01 compared to the siRNA negative control group, determined by unpaired t-test with the Mann–Whitney test.

**Figure 7:**
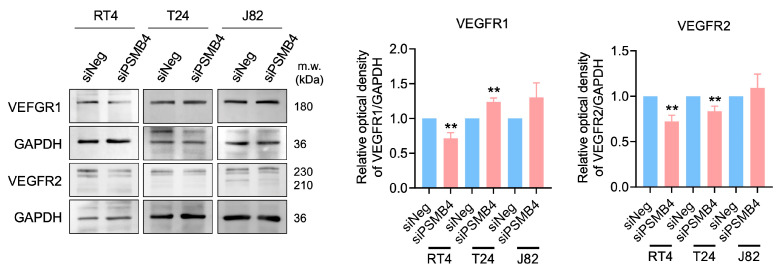
The expression of angiogenesis-related proteins in HUVECs after the silencing of PSMB4. The conditioned media of RT4, T24, and J82 cells transfected with siPSMB4 were collected. HUVECs were cultured with these conditioned media for 6 h. The protein levels of VEGFR1 and VEGFR2 in HUVECs after treatment with conditioned medium were measured by Western blotting. GAPDH was used as the loading control. Data are presented as the mean ± SEM (*n* = 5). ** *p* < 0.01 compared to the siRNA negative control group, determined by unpaired t-test with the Mann–Whitney test.

The migration of endothelial cells leads to the formation of the blood vessel lumen [17,18]. The wound healing assay showed that the conditioned medium from siPSMB4 cultures decreased HUVEC migration ability compared with the control group (Figure 6). We also analyzed angiogenesis-related protein expression in HUVECs. The expression of VEGFR1 was reduced in RT4 cells but elevated in T24 cells, and VEGFR2 expression was decreased in RT4 and T24 cells (Figure 7).

### 2.5. Knockdown of PSMB4 in Bladder Cancer Cells In Vivo

**Figure 8:**
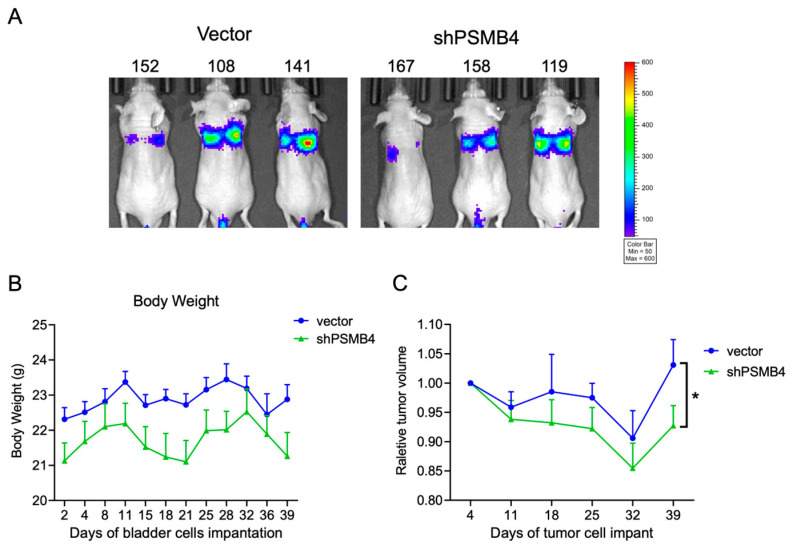
Effect of PSMB4 knockdown on lung metastasis in a bladder cancer xenograft mouse model. Nude mice were intravenously injected with T24 cells with PSMB4 knockdown, and tumors were allowed to grow for 3 weeks. (**A**) The effects of the control shRNA and shPSMB4 on tumor size were observed using bioluminescence imaging with an in vivo imaging system (IVIS). (**B**) The body weights were measured twice a week. (**C**) The relative volumes of vector and shPSMB4 tumors. Data are presented as the mean ± SEM (*n* = 8 for each group). * *p* < 0.05 compared to the vector group, determined by unpaired t-test with the Mann–Whitney test.

To clarify the role of PSMB4 in vivo, we established a bladder cancer metastasis model in nude mice by intravenous injection to evaluate the effect of PSMB4 knockdown on metastasis (Figure 8A). Body weight fluctuations were unaffected in each group (Figure 8B). After 39 days, the relative volumes of lung tumors were smaller in the shPSMB4 group than in the vector control group (Figure 8C). These results suggest that PSMB4 may regulate bladder cancer progression, but the detailed mechanism still needs to be confirmed.

## 3. Discussion

This study examined the role of PSMB4 in bladder cancer. After siPSMB4 transfection, the viability of RT4 cells was decreased at 72 h, a difference that was not observed in T24 and J82 cells. Although silencing PSMB4 reduced the migration ability of human bladder cancer cells, different signals may be involved in low- and high-grade bladder cancers. Moreover, knockdown of PSMB4 inhibited HUVEC tube formation, possibly due to the reduction in the VEGF-B content in the conditioned medium collected from bladder cancer cells. These findings may provide new potential targets for the clinical treatment of human bladder cancer.

PSMB4 inhibition prevented the migration of human bladder cells, possibly due to the decreases in FAK and MLC expression. In our previous study, the levels of adhesion-associated proteins, including p-FAK, p-paxillin, integrin β1, and integrin β3, were reduced after knockdown of PSMB4 expression in LN229 human glioblastoma cells [13]. In human breast cancer research, activation of PI3K-Akt and subsequent phosphorylation of downstream FAK was found to increase the migration of cancer cells [19]. In a study of head and neck squamous cell carcinoma, integrin can regulate the activity of paxillin and downstream Rac1 through the FAK/Src complex to complete the cell migration process [20]. In addition, the proteasome inhibitor MG132 inhibits tumor development by inactivating NF-κB signaling, synergizes with paclitaxel (PTX) to enhance antitumor activity, induces apoptosis and G2 arrest, and inhibits viability and migration to inhibit tumor progression [21]. These results suggest that adhesion-related proteins may regulate the migration ability of human bladder cancer cells after PSMB4 inhibition.

PSMB4 inhibition reduces HUVEC tube formation, possibly due to the decreased secretion of VEGF from bladder cancer cells. The members of the VEGF family, namely, VEGF-A, -B, -C, -D, and -E, mediate their signaling pathways through interactions with VEGF receptors. VEGFR-1 has the high affinity for VEGF-A, VEGF-B, and placental growth factor. VEGF-B has been reported to regulate cell viability through the MAPK and PI3K/AKT cascades [22]. Moreover, VEGF-B is involved in controlling the diameter and size of vessels [23]. Both VEGF-C and VEGF-D can bind to VEGFR-2 and VEGFR-3 [24,25]. VEGF-C is recognized as the fundamental promotor of the proliferation and migration of lymphatic endothelial cells [26]. In our results, the decrease in VEGF-B, but not VEGF-A, -C, and -D, in PSMB4-knockdown bladder cancer cells was verified. In addition, the content of VEGF in the conditioned medium was reduced, leading to the inhibition of tube formation.

There are limitations in this study. Although primary cells from patients were not used, a bladder cancer tissue array was used for HE and IHC staining to show that the expression of PSMB4 was higher in cancer tissues than in adjacent normal tissues. In the future, obtaining bladder cancer cells derived from patients to confirm the inhibitory effects of PSMB4 on growth, migration, and angiogenesis will provide further evidence supporting the role of PSMB4 in bladder cancer.

PSMB4 inhibition prevented the migration of HUVECs, which may be due to the decrease in VEGF receptor expression. Previous research indicates that widespread VEGFR-1 and VEGFR-2 expression on the vascular endothelium contributes significantly to angiogenesis [27]. The natural medicinal product auriculasin potently inhibits VEGF-induced chemotactic cell migration and capillary-like structure formation, and its properties target the PI3K/AKT/mTOR, MAPK, and VEGFR2 pathways in HUVECs [28]. Therefore, VEGF and angiogenesis-related factors play important roles in the angiogenic ability of bladder cancer. This research shows that PSMB4 effectively inhibits angiogenesis by regulating VEGF-receptor-related signaling pathways, further verifying the great clinical potential of PSMB4 in combination with targeted antiangiogenic therapy.

## 4. Materials and Methods

### 4.1. Cell Culture

A bladder carcinoma tissue microarray (BL802b) was purchased from Biomax (Derwood, MD, USA). The human bladder cancer cell lines RT4 (HTB-2) and T24 (HTB-4) were purchased from American Type Culture Collection (ATCC, Manassas, VA, USA) and cultured with McCoy’s 5A medium. J82 cells (ATCC HTB-1) were maintained in DMEM (Gibco, Billings, MT, USA) containing 10% fetal bovine serum (CORNING, Corning, NY, USA), 1 mM sodium pyruvate and 1 mM L-glutamate (Thermo Fisher Scientific, Waltham, MA, USA). Human umbilical vein endothelial cells (HUVECs, H-UV001) were purchased from Bioresource Collection and Research Center (BCRC, Hsinchu, Taiwan) and cultured with endothelial cell medium (ECM, Cat, No. 1001, ScienCell Research Laboratories, Carlsbad, CA, USA) containing 5% fetal bovine serum (FBS, Cat. No. 0025), endothelial cell growth supplement (ECGS, Cat. No. 1052), and 1% antibiotic solution (P/S, Cat. No. 0503). The cells were incubated in a humidified atmosphere containing 5% CO_2_ at 37 °C.

### 4.2. siRNA Transfection

RT4, T24, and J82 human bladder cancer cells were seeded in 6-well plates at 3 × 10^5^ cells per well. The cells were transfected with siNegative control (siNeg, siGENOME Control Pool 50 nM, Catalog ID:D-001206-13-05; Dharmacon Horizon Discovery, Cambridge, UK) or siPSMB4 (Cat. No. M-011362-00-0010) using Lipofectamine 3000 reagent (Cat. No. L3000001, Thermo Fisher Scientific, Waltham, MA, USA). The cells were used for further experiments after 72 h of transfection.

### 4.3. MTT Assay

RT4, T24, and J82 human bladder cancer cells were seeded in 96-well plates at 1 × 10^3^ cells per well. After 72 h of transfection with siPSMB4, the survival status of the PSMB4-inhibited group was compared to that of the control group. MTT reagent (3-(4,5-dimethyl-2-thiazolyl)-2,5-diphenyl-2H-tetrazolium bromide) (M6494, Thermo Fisher Scientific, Waltham, MA, USA) was added to each well for 3 h, then the cells were lysed with DMSO. The optical absorbance was measured with an ELISA plate reader (Synergy HTX, BioTek, Winooski, VT, USA) at 570 nm.

### 4.4. Colony Formation Assay

One hundred RT4, T24, or J82 human bladder cancer cells were plated in 6-well culture dishes, and the medium was changed every two days. The cells were treated with siNeg or siPSMB4 to evaluate the long-term survival status. After 10 days of culture, cells were fixed with 10% paraformaldehyde and stained with Coomassie Brilliant Blue G250 (Thermo Fisher Scientific, Waltham, MA, USA). Then, the colonies in each well were photographed and counted.

### 4.5. Wound Healing Assay

PSMB4 was inhibited via siRNA transfection for 72 h in human bladder cancer cell lines (RT4, T24, J82). Conditioned medium (CM) from PSMB4-knockdown cells was added to HUVECs. Wounds were made with a 200 μL pipette tip and were observed and imaged both at 0 h and at 6 h. Then, the difference in the wound area was calculated with ImageJ software (version 1.49, National Institutes of Health, Bethesda, MD, USA).

### 4.6. Western Blotting

Human bladder cancer cell lines (RT4, T24, and J82) were treated with siNeg or siPSMB4 for 72 h, then lysed with lysis buffer (GE Healthcare, Chicago, IL, USA) and homogenized using a Qsonica Q700 ultrasonic cell disruptor. The protein concentration was determined with a protein assay kit (Bio-Rad Life Science, Hercules, CA, USA). A total of 60 μg of protein was loaded into each well of a 10% SDS-PAGE gel. The proteins in the samples were transferred onto nitrocellulose membranes after separation by electrophoresis. The membranes were blocked with BlockPRO1 Min Protein-Free Blocking Buffer (Visual Protein, Taipei, Taiwan). The proteins to be analyzed were focal adhesion kinase (FAK, 71433S), integrin b1 (34971S), MLC (8505S), p-MLC (3671S), VEGF-C (2445S), VEGFR2 (2479S), GAPDH (2118S) (antibodies obtained from Cell Signaling, Danvers, MA, USA), PSMB4 (ab137067), p-FAK (ab39967), VEGFR1 (ab32152) (antibodies obtained from Abcam, Waltham, Boston, USA), and integrin b3 (611141, BD Biosciences, Franklin Lakes, NJ, USA). The primary antibodies were diluted in blocking buffer at 4 °C for more than 16 h. Afterward, the nitrocellulose membrane was washed several times with TBS-Tween buffer and then incubated with the secondary antibodies (horseradish-peroxidase-conjugated goat anti-mouse or rabbit IgG (Biolegend, San Diego, CA, USA)) diluted 1:5000 at room temperature for 1 h. Then, the nitrocellulose membranes were visualized with chemiluminescence reagent (Bio-Rad Life Science, Hercules, CA, USA) and imaged with a luminescence imaging CCD camera system. The results were analyzed with Image Lab software from Bio-Rad Laboratories (Version 6.1).

### 4.7. Tube Formation Assay

PSMB4-knockdown-cell conditioned medium was added to 1.1 × 10^4^ HUVECs; then, the mixture was added into the wells with Matrigel (Corning, Arizona, US, Cat. 356235) and the angiogenesis factors VEGF-121, VEGF-165, and VEGF-B (2 µg/mL) were added (Cat. No.: HY-P7420, HY-P7110A, and HY-P72431, MedChem Express, Monmouth Junction, NJ, USA). After culture for 6 h, images were captured and the length of vessels and the number of vessel branch points were quantified and analyzed with AngioTools.

### 4.8. Enzyme-Linked Immunosorbent Assay (ELISA)

The ELISA kit for VEGF was purchased from R&D Systems (DY293B-05, Minneapolis, MN, USA), and the concentration of VEGF in the culture medium was measured according to the manufacturer’s instructions. The values were read and corrected with an ELISA plate reader, and the results are expressed in pg/mL.

### 4.9. RNA Extraction and Real-Time Polymerase Chain Reaction (RT-PCR)

RT4, T24, and J82 human bladder cancer cells were transfected with siNeg or siPSMB4. After transfection, RNA was extracted using a GENEzol TriRNA Pure Kit (GZX100, Geneaid Biotech, New Taipei City, Taiwan) and reverse transcription (RR037B, TAKARA, Kusatsu-Shi, Shiga, Japan) was performed with a T100 Thermal Cycler to convert approximately 500 ng of RNA to DNA. Gene expression was analyzed on a LightCycler480 system (Roche, Mannheim, Germany). PSMB4: forward 5′-ACTGGTTATGGTGCATACTTG-3′ and reverse 5′-TTGTAAGAACGGGCATCTC-3′; VEGF-B: forward 5′- AAGGACAGTGCTGTGAAGCCAG-3′ and reverse 5′- TGGAGTGGGATGGGTGATGTCA-3′; VEGF-C: forward 5′- GCCAATCACACTTCCTGCCGAT-3′ and reverse 5′- AGGTCTTGTTCGCTGCCTGACA-3′.

### 4.10. Short Hairpin RNA (shRNA) Transfection

The TRC2 vector and pLKO-shPSMB4 (ASN0000000003 and TRCN0000273111) were purchased from the National RNAi Core Facility at Academia Sinica in Taiwan. 293T cells were transfected with shPSMB4, pCMV-ΔR8.9, and pMD2.G to produce lentiviruses. T24 cells were seeded in 6-well plates, cultured to 95% confluence, and infected with lentivirus in the presence of polybrene (8 μg/mL) (Thermo Fisher Scientific, Waltham, MA, USA). The next day, the T24 cell medium was replaced with McCoy’s 5a medium containing puromycin (2 μg/mL). Cell lines were maintained in a humidified atmosphere at 37 °C and 5% CO_2_. The sequences of the shRNAs were as follows. Scramble (ASN0000000003) 5′-CCGGCCTAAGGTTAAGTCGCCCTCGCTCGAGCGAGGGCGACTTAACCTTAGGTTTTT-3′ and shPSMB4 (TRCN0000273111) 5′- CCGGTACCGAGATGCCCGTTCTTACCTCGAGGTAAGAACGGGCATCTCGGTATTTTTG-3′.

### 4.11. Bladder Cancer Xenograft Animal Models

All mouse experiments were approved by the laboratory animal center of National Defense Medical Center, Taiwan (IACUC No. 22-280). BALB/cAnN.Cg-Foxnlnu/CrlNarl nude mice (8 weeks old, male) were provided by the National Laboratory Animal Center (NLAC), Taipei, Taiwan. A total of 1 × 10^6^ shPSMB4 T24-Luc cells were injected into the mouse circulatory system through the tail vein. The animals were divided into two groups (weight, approximately 20–25 g; *N* = 5 mice in each group): 1. scramble control group and 2. PSMB4 inhibition group. A noninvasive in vivo imaging system (IVIS) was used to observe and analyze the growth of tumors once a week, and the body weights of the mice were recorded twice a week. After 39 days, the animals were sacrificed and the lung tissues were analyzed for the expression of metastasis-related proteins.

### 4.12. Statistical Analysis

All experiments were carried out at least three times, and the results are expressed as the sum of the means plus or minus the standard error (mean ± SEM). One-way ANOVA was used to detect whether there were significant differences between groups. In all analyses, *p* < 0.05 indicated a statistically significant difference.

## 5. Conclusions

Inhibition of PSMB4 resulted in a decrease in the migration ability of human bladder cancer cells through a reduction in the expression of the adhesion-related proteins FAK and MLC. Knockdown of PSMB4 decreased VEGF-B secretion and angiogenesis in human bladder cancer. PSMB4 inhibition also decreased the migration ability of and the expression of the angiogenesis-related protein VEGFR2 in HUVECs. The PSMB4 knockdown decreased the relative volumes of lung tumors in the animal metastasis model. These findings indicate that PSMB4 may serve as a potential target for therapeutic strategies in human bladder cancer.

## Data Availability

All of the data produced or analyzed during this work are presented in full in this published paper and its Supplementary Information Files or, upon reasonable request, are available from the corresponding author.

## Supplementary Materials

The following supporting information can be downloaded at: https://www.mdpi.com/article/10.3390/ijms25105559/s1.
