# Supplementary material for: The Reduction of PSMB4 in T24 and J82 Bladder Cancer Cells Inhibits the Angiogenesis and Migration of Endothelial Cells
Source: Int J Mol Sci. 2024 May 20;25(10):5559. doi: 10.3390/ijms25105559 (PMC11122396; doi:10.3390/ijms25105559)
Supplement: Supplementary file 1 [file ijms-25-05559-s001.zip › ijms-3007112-supplementary.pdf]

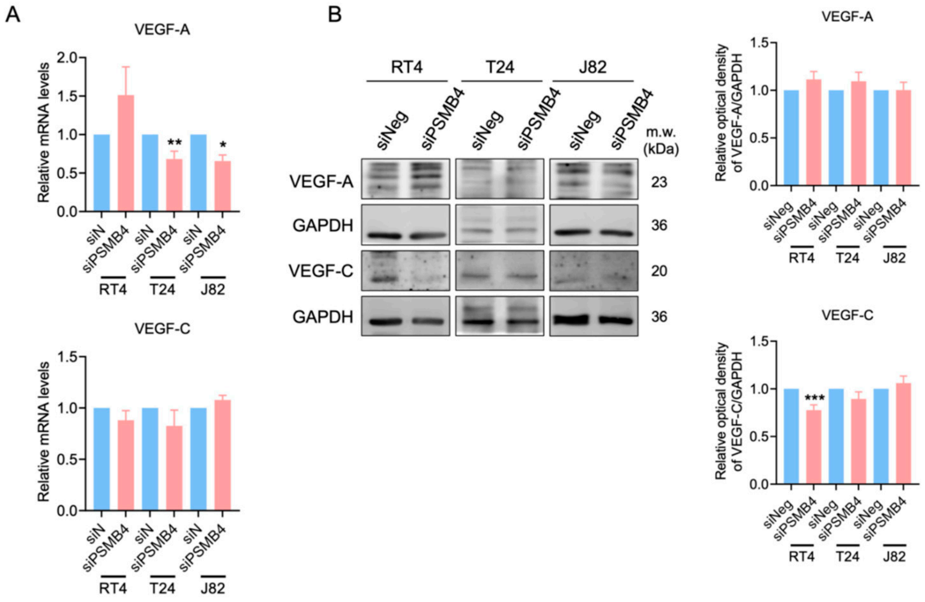

Supplementary Figure S1. The expression of angiogenesis-related proteins in bladder cancer cells after silencing of PSMB4. (**A**) The mRNA levels of VEGF-A and VEGF-C in RT4, T24 and J82 cells after treatment with siPSMB4 for 72 hours were measured by real-time PCR. (**B**) VEGF-A and VEGFC protein expression after siPSMB4 transfection for 72 hours in RT4, T24 and J82 cells was measured by Western blotting. * p<0.05, ** $p < 0.01$, and *** $p < 0.001$ compared to the siRNA negative control group. GAPDH was used as the loading control.
